# Have Studies that Measure Lumbar Kinematics and Muscle Activity Concurrently during Sagittal Bending Improved Understanding of Spinal Stability and Sub-System Interactions? A Systematic Review

**DOI:** 10.3390/healthcare6030112

**Published:** 2018-09-08

**Authors:** Alister du Rose

**Affiliations:** Faculty of Life Sciences and Education, University of South Wales, Treforest, Pontypridd, Wales CF37 1DL, UK; alister.durose@southwales.ac.uk

**Keywords:** spinal stability, spinal motion, electromyography, low back pain, flexion

## Abstract

In order to improve understanding of the complex interactions between spinal sub-systems (i.e., the passive (ligaments, discs, fascia and bones), the active (muscles and tendons) and the neural control systems), it is necessary to take a dynamic approach that incorporates the measurement of multiple systems concurrently. There are currently no reviews of studies that have investigated dynamic sagittal bending movements using a combination of electromyography (EMG) and lumbar kinematic measurements. As such it is not clear how understanding of spinal stability concepts has advanced with regards to this functional movement of the spine. The primary aim of this review was therefore to evaluate how such studies have contributed to improved understanding of lumbar spinal stability mechanisms. PubMed and Cochrane databases were searched using combinations of the keywords related to spinal stability and sagittal bending tasks, using strict inclusion and exclusion criteria and adhering to PRISMA guidelines. Whilst examples of the interactions between the passive and active sub-systems were shown, typically small sample sizes meant that results were not generalizable. The majority of studies used regional kinematic measurements, and whilst this was appropriate in terms of individual study aims, the studies could not provide insight into sub-system interaction at the level of the spinal motion segment. In addition, the heterogeneity in methodologies made comparison between studies difficult. The review suggests that since Panjabi’s seminal spinal control papers, only limited advancement in the understanding of these theories has been provided by the studies under review, particularly at an inter-segmental level. This lack of progression indicates a requirement for new research approaches that incorporate multiple system measurements at a motion segment level.

## 1. Introduction

Spinal stability was interpreted by Panjabi (1992) to be dependent on the highly co-ordinated and optimised interactions between three sub-systems, the passive (ligaments, discs, fascia and bones), the active (muscles and tendons) and the neural control systems. According to this theory, if there is dysfunction within a specific system, compensation may be provided by adaptations in the other systems [1,2]. Panjabi suggested that abnormally increased muscle activation is a stabilisation mechanism compensating for a loss of spinal stability, a theory repeatedly supported in the subsequent literature. It has also been suggested that extended periods of myoelectrical silence (i.e., flexion relaxation) during prolonged flexion can result in a loss of spinal stability [3], and that such prolonged flexion also results in a transfer of extension moment between passive tissues and spinal muscles [4]. Such adaptations have been proposed as possible precipitators of low back pain (LBP), and it has been shown that trunk muscle recruitment patterns can be different between healthy and low back pain populations [5]. Investigations addressing the role of the active system are frequently limited however due to the inherent heterogenity of electromyography (EMG) signal data [6].

In order to improve understanding of the complex interactions between sub-systems, and possible biomechanical relationships with LBP, it is necessary to take an approach that incorporates the measurement of both lumbar kinematic and trunk muscle activation data [7]. Studies that do so include investigations into sub-system changes in response to pertubation [8], how such responses are influenced by paraspinal muscle fatigue [9,10,11] and the effect of spinal creep deformation [12]. A recent systematic review of such studies however, suggests that whilst a mechanism to achieve spinal stability may be for the central nervous system (CNS) to generate early postural muscle activity, this occurs regardless of the level of fatigue [13]. It was also concluded that spinal tissue creep likely does not influence spinal stability in the context of purtubation, and that in both cases, the high methodological heterogeneity between studies meant that comparison between studies was difficult [13]. In addition, as controlled pertubation studies investigate responses around a neutral spine position, no insight can be gained into possible sub-system interactions during the entire range of spinal movement.

In terms of the investigation of movements in the sagittal plane, the study of the Flexion Relaxation Phenomenon (FRP) [14] is another possible way in which insight into sub-system interaction can be gained. The deactivation of paraspinal muscle activity during the final stages of forward flexion has been interpreted as the transfer of moment between the active and passive sub-systems [15], and feasibly provides an insight into sub-system interaction. It has therefore been extensively studied [16,17,18,19], however the majority of studies only incorporate the measurement of regional kinematics, and therefore do not provide any insight from the level of the motion segment [20]. It could be argued that investigations at the inter-vertebral level are important, as inter-system feedback mechanisms are believed to act at this level [21].

It has also been common for studies to focus on individual systems in isolation, in an attempt to relate changes within each system to conditions such as LBP. Indeed, in terms of the active system, LBP has been associated with changes in paraspinal muscle cross sectional size [22], activation timings [23,24] and muscle activation amplitudes [5,25,26,27,28]. Focus on the passive system has shown potential links between LBP and lumbar range of motion (ROM) [7,29,30,31,32,33], and postural parameters [34], however such investigations, by considering kinematic or muscle activity parameters in isolation, can only speculate as to how such changes may relate to adapatations in the other sub-systems.

In addition, many of these studies have produced conflicting results, and there is therefore an argument that attempts should first be made to improve understanding of normal (i.e., spinal biomechanical behaviours of non-LBP populations), so as to better understand what is abnormal [35]. Investigations of the kinematics of normal controls has shown how regional spinal ranges of motion may be associated with the ranges achieved in another spinal region (i.e., as one region moves more there may be less movement in another region) [36,37], however again, such adaptations again cannot be explained in terms of sub-system adaptation, as only a single system was considered.

The complexity and inaccessability of investigating spinal control mechanisms makes the interpretation of study findings difficult. A key problem is that sub-system interaction is dynamic, and therefore the study of two or more systems concurrently in living humans requires instrumentation that can do so dynamically and concurrently. Physical activities involving sagittal bending are commonplace activities of daily living [38], and as the most widely investigated functional task, an improved knowledge of sub-system interactions during sagittal bending would be of clinical interest. Currently there are no reviews of studies that have investigated dynamic sagittal bending movements using a combination of EMG and lumbar kinematic measurements, and as such it is not clear how understanding of in vivo spinal stability concepts has advanced as a result of investigations into this functional movement of the spine. It is also of interest how the findings of such studies have helped distinguish between low back pain and non-low back pain populations.

This review addressed two fundamental questions. (1) How have studies that combine concurrent lumbar kinematic and muscle activity measurements during sagittal bending improved understanding of lumbar spinal stability mechanisms (i.e., sub-system interactions)? (2) Are studies that combine concurrent lumbar kinematic and muscle activity measurements during sagittal bending able to distinguish between groups of healthy controls (i.e., no low back pain) and those with low back pain?

## 2. Methods

### 2.1. Literature Search Strategy

PubMed and the Cochrane library were searched in March and April 2017. The systematic search was performed using combinations of the following keywords: (Electromyography or EMG or Flexion Relaxation or FRP and Kinematics or Range of Motion or ROM and Low Back Pain or Lumbar Spine and Flexion or Bending and Stability or Stabilization). Article screening was conducted by the author and was restricted to English publications between 1992 and 2017 in order to only include articles post Panjabi’s seminal papers that originally explored the theory of sub-system interactions [1,2].

### 2.2. Inclusion and Exclusion Criteria

**Figure 1:**
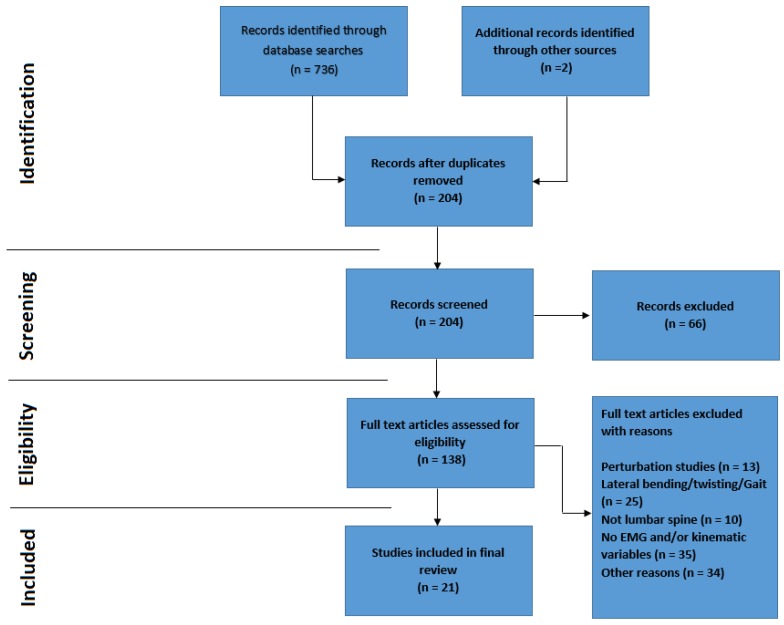
Prisma flowchart. Note: Additional articles (n = 2) were sourced via a manual search through the reference lists of the articles identified in the database search.

Articles were included for review if they met the following inclusion and exclusion criteria. Inclusion criteria consist of (1) Studies must be in vivo using adult participants (2) Weight-bearing movement in the sagittal plane (3) Include both EMG (including the lumbar paraspinal muscles) and lumbar kinematic measurements (4) Relate study findings to stability theories or spinal stabilisation. Exclusion criteria included (1) Pertubation studies (as the articles of interest were to include active movement (2) Studies measuring creep or fatigue (as single cycles of dynamic tasks will unlikely result in either) (3) Studies not investigating the lumbar spine specifically (i.e., cervical, thoracic or shoulder) (4) Studies investigating lateral flexion, axial rotation or gait (i.e., not including sagittal flexion) (5) Non-human studies (e.g., feline studies) (6) Repeatability trials. A flowchart outlining the citation selection process is shown in Figure 1. Other reasons for study exclusion included manipulation by design (e.g., investigations into the effects of noxious stimuli, high heels, taping, and exercise).

### 2.3. PRISMA (Preferred Reporting Items for Systematic Reviews and Meta-Analyses)

This systematic review adheres to the PRISMA guidleines.

### 2.4. Study Quality Assessment

This review uses a quality assessment tool developed by Abboud et al. [13] that was adapted from the Quality Index of Downs and Black [39]. Abboud et al. also created an assessment designed to specifically interpret the quality of studies incorporating EMG, which was based on Surface ElectroMyoGraphy for the Non-Invasive Assessment of Muscles (SENIAM) [40] and International Society of Electrophysiology and Kinesiology (ISEK) guidelines. This novel assessment was also incorporated. The quality assessment of each paper was performed twice (approximately 12 months apart) by a single reviewer (*r* = 0.98).

#### 2.4.1. Overall Quality Assessment

The original quality index developed by Downs and Black (1998) has been shown to have good test-retest (*r* = 0.88) and inter-rater observability (*r* = 0.75) [13]. The adapted tool consists of 10 items that were deemed appropriate for the purpose of this review. The items included the following questions (1) Is the hypothesis/aim/objective of the study clearly described? (2) Are the main outcomes to be measured clearly described in the Introduction or Methods section? (3) Are the characteristics of the patients included in the study clearly described? (4) Are the interventions of interest clearly described? (5) Are the main findings of the study clearly described? (6) Does the study provide estimates of the random variability in the data for the main outcomes? (7) Have actual probability values been reported (e.g., 0.035 rather than <0.05) for the main outcomes except where the probability value is less than 0.001? (8) Were those subjects who were prepared to participate representative of the entire population from which they were recruited? (9) If any of the results of the study were based on “data dredging”, was this made clear? (10) Were the statistical tests used to assess the main outcomes appropriate? All items were scored either 0 or 1. This produced a total quality score out of 10 for each study, with the exception of those articles that did not require population comparison, and so were scored out of 9 (Table 1). Final scores were converted into percentages and combined with the EMG quality scores, providing an overall impression of study quality (Table 2).

#### 2.4.2. Specific EMG Quality Assessment

The checklist developed by Abboud et al. (2017) [13] consists of 12 items divided into 4 sections (Table 2). The first section considers the use of surface electromyography (sEMG) electrodes (as all studies reviewed used sEMG and none used needle EMG, despite the inclusion of both terms in the literature search) and comprises a score for inter-electrode distance, electrode material and construction (i.e., bipolar). The second section considers participant skin preparation, the use of reference electrodes and electrode placement and fixation. The third section considers signal processing and includes items regarding the use of filters, rectification methodology, sampling and processing. The final section considers the appropriate use of normalisation. Each item was scored 0 or 1, and a score of 1 was attributed to a section if the item totals reached 2 or more. This produced an EMG quality score out of 4 for each study, with the exception of those articles where normalisation was not deemed necessary, and so were scored out of 3. These scores were also converted into percentages and combined with the study quality assessment scores above (Table 3).

## 3. Results

Out of a total of 736 articles identified through the literature search, 21 satisfied the inclusion/exclusion criteria. The screening process is outlined in the PRISMA flowchart (Figure 1).

### 3.1. Overall and EMG Quality Assessment

The overall quality assessment scores ranged from 44–100% with a mean total score of 80% (Table 1). All of the selected studies scored a 1 for their descriptions of methodology and study findings. The studies also performed well in terms of the quality of hypothesis and outcome descriptions (19/21 and 20/21 respectively), and their use of appropriate statistics and absence of data dredging (both 20/21). Areas in which the studies generally scored poorly included the description of participant characteristics (9/21) and the reporting of actual probability values (7/21). The EMG quality assessment showed scores ranging from 25–100% with a mean total score of 73% (Table 2). The assessment showed that the majority of EMG studies adequately reported the normalisation and signal processing elements, however it also highlighted a mixture of study quality when considering the detail of electrode use. The combined overall and EMG quality index scores ranged from 47–100% with a mean total score of 77% (Table 3).

### 3.2. General Characteristics of the Reviewed Studies

All of the studies reviewed could be placed into one of 4 categories, the majority being studies relating in some way to the flexion relaxation phenomenon (FRP): [15,16,17,20,26,43,45,47,49,51,52,53], or comparisons between LBP and healthy control participant groups: [6,7,17,20,26,42,48,49,51,53]. There was a degree of crossover however as some comparison studies also incorporated the FRP. Other study areas included EMG activation studies (other than FRP) [6,7,26,35,42,44,46,48,50,53], and spinal modelling [6,15,41,52].

Table 4 shows that typically regional kinematics were measured, with the exception of the inter-vertebral methodology used by Kaigle et al. (1998) [20]. Indeed the methods used to measure regional ROM varied between studies. This trend was also apparent in terms of electrode positioning, with many different sites being used to record activity from the same designated muscle. The table also highlights the generally small sample sizes used in this type of study, with the majority using fewer than 30 participants. The only exceptions were the studies of Mayer et al. (2009) [49], Kienbacher et al. (2016) [47], Lariviere et al. (2000) [6] and Neblett et al. (2003) [51] with participant numbers of 134, 216, 33 and 66 respectively.

Whilst the reliability of kinematic measurements was not established in all of the reviewed studies, in those that did, reliability was typically found to be excellent, however different approaches to determine reliability were evident. Dankaert et al. (2009) for example determined the inter-trial reliability of the 3 Space Fastrak system (Polhemus Navigation Science Division, Kaiser Aerospace, VT), using intraclass correlation coefficients (ICC)(3,1) [7]. Inter-trial reliability was shown with ICC’s of 0.85 or greater and standard error of measurement (SEM) was also included. This was in contrast to Neblett et al. (2003) who used Pearson’s product moment correlations, showing inter and intra examiner reliability of the inclinometers used to be *r* = 0.92 or greater [51].

### 3.3. Comparing Healthy Control and Low Back Pain Groups

Of the studies above comparing LBP and healthy control groups, the majority found objective differences between the groups. Burnett et al. (2004) [42]: showed that the LBP group had greater lower lumbar flexion and reduced multifidus activity compared to controls, whilst controls showed greater upper lumbar flexion. In Dankaerts et al.’s study 2009 [7], differences were found in terms of multifidus activity and spinal kinematics between both flexion pattern (FP) and active extension pattern (AEP) provocation sub-groups and healthy controls. In summary, multifidus activity was increased in the AEP group relative to the FP at the end of flexion, and the FP group demonstrated increased activity compared to the healthy controls. These patterns were attributed to the maintenance of the lumbar lordosis during flexion in the AEP group, and the similar spinal curvature between FP and healthy control groups. The Kaigle study provided the only inter-vertebral insight into active and passive system interactions, using intra-osseous pins connected to a sliding linkage transducer system to measure inter-vertebral angular rotation [20]. The study showed that inter-vertebral angular range was significantly smaller in the LBP group, and that the majority of patients showed no reduction in paraspinal muscle activity at the end ranges of flexion. Indeed, the FRP was only present in participants who demonstrated near complete inter-vertebral rotation before maximum global trunk flexion was attained.

Two of the studies were linked and provided similar conclusions. Neblett et al. (2003) [51] showed that in terms of the FRP and patients, all LBP patients that underwent a rehabilitation program achieved normal ROM, and subsequently demonstrated the FRP, whilst Mayer et al., 2009 [49] likewise concluded that normal lumbar ROM appears to correlate with the FRP and was therefore absent in many LBP participants. However, both FRP and ROM measurements responded well after a generic rehabilitation program.

Using a network modelling and analysis approach, Liu et al. (2011) [48] claimed to be able to clearly distinguish LBP and healthy control participants using symmetric patterns and network features, and Paquet et al. (1994) [53] showed that when flexion was performed over the same rate and range, LES activity was significantly greater in the LBP group. Participants in the study with an absent FRP also demonstrated increased ROM of the hip around full flexion.

Not all studies demonstrated an ability to differentiate between LBP and control groups however. Lariviere et al. (2000) [6] for example used a novel principal component analysis (PCA) technique to investigate whether EMG and kinematics could distinguish between the two. Their PCA analysis consisted of two steps. Firstly using EMG activity envelopes from control subjects, a reference model was developed (i.e., a criteria for normal). Secondly ‘distance measures’ were calculated relative to the reference model. The EMG waveform of a participant was labelled as abnormal if the ‘distance value’ was outside a 95% confidence interval calculated from the control subjects. Whilst being sensitive to trunk ROM, the distance measures were not sensitive to low back pain status. The authors argued that this was likely due to the relatively small sample size, and therefore inadequate considering the large heterogeneity control populations. In conclusion it was considered that the tool developed was not useful in terms of distinguishing between LBP patients and controls. Sanchez-Zuriaga et al. (2015) [26] also demonstrated contrasting results, as the authors found no significant difference between LBP and healthy groups, in either FRP or lumbar ROM. The study did however show significantly greater LES activity in LBP participants during the flexion-extension task, and the LBP patients were participating during a pain free period.

### 3.4. Flexion Relaxation Studies

The results of some of the FRP studies [15,20,49,51,53] have already been mentioned. Callaghan and Dunk (2002) showed that during slumped sitting the TES exhibited the FRP, but the LES did not. The authors also demonstrated that this deactivation occurred earlier (i.e., at a smaller lumbar flexion angle) than LES deactivation during flexion from standing [43]. In contrast to these findings, O’Sullivan et al. (2006) showed that although LMU activity decreased (i.e., FRP was present) when going from a neutral to a slumped seated position, there were varying patterns in TES activity, as approximately half the participants showed an increase in activity and half a decrease [17]. Hashemirad et al. showed that trunk flexibility can influence FRP, with greater flexibility relating to FRP onset at larger flexion angles [45], and Luhring et al. (2015) [16] chose to address the problem of using different methodologies to measure regional kinematics in FRP studies (by acknowledging a wide range of normalised and un-normalised FRP onset angles), investigated whether lumbar (i.e., T12-L5) or trunk (i.e., shoulders and hips) angles were more consistent in terms of EMG cessation and onset. The study found that lumbar kinematic measurements were more consistent.

Finally, the study conducted by Ning et al. (2012) [52] suggested that passive tissues can produce significant loads at earlier trunk flexion angle than previously believed i.e., those suggested by Kaigle et al. (1998) [20] where erector spinae deactivation was shown to begin at between 71° and 77° of grouped inter-vertebral level flexion, or Peach et al. (1998) [35] where FRP was shown to occur between 60° and 70°.

### 3.5. Models

Arjmand et al. (2010) [41] compared EMG-driven (EMGAO) and multi-joint Kinematics-driven (KD) models in terms of muscle force and spinal load estimation. During a flexion task the KD model predicted greater paraspinal muscle activity compared to the EMGAO model and therefore shear and compression forces were also higher. Predictions made using the EMGAO model were also found to be level specific (i.e., L5-S1), and could not be an accurate representation of other lumbar levels (Arjmand et al. (2010) [41]). Ning et al. (2012) [52] as discussed above, determined at what trunk flexion angle the passive tissues were able to generate a significant extensor moment during forward bending (Ning et al. (2012) [52]), and McGill and Kippers 1994 [15] showed that although paraspinal muscles are electrically silent at the end range of forward flexion, these muscle continue to provide elastic resistance via passive stretching.

## 4. Discussion

### 4.1. Quality Assessment

The mean of the combined quality check and EMG scores was 77%, suggesting that the overall quality of the studies reviewed was generally good. Of particular note were the studies of Dankaerts et al. (2009) [7], Kienbacher et al. (2016) [47] and O’Sullivan et al. (2006) [17], which all scored 100%. The majority of studies used muscle activity amplitude as their key EMG parameter, and it was apparent that the majority also reported the relevant normalisation technique. The high percentage of good scores in this area, therefore makes it easier to compare amplitude results between studies. Other areas of apparent good quality reporting included the descriptions of the hypothesis, aims, and objectives of the studies, the main outcomes to be measured, the interventions of interest and the main findings. In terms of EMG quality, relevant signal processing information was also usually well reported.

This high standard of reporting was not evident throughout the review however, and trends in areas that were weaker emerged. In terms of the Quality Index assessment scores, the reporting of participant characteristics (including inclusion and exclusion criteria) and actual probability values was poor, with over half of all studies included scoring zero for these categories. Regarding the EMG quality assessment scores there was notably poor reporting of skin preparation techniques, the placement and fixation of electrodes and details regarding the use of reference electrodes, information that would be important if these studies were to be replicated. Sample sizes were also generally small, with 17/21 studies using samples of <30 participants. This potentially weakens the statistical power of these studies and increases the chance of Type II errors.

### 4.2. Spinal Stability and Sub-System Interaction

Whilst the studies under review do consider spinal control mechanisms, with only a single study providing inter-vertebral information, discussions concerning possible mechanisms at the motion segment level were limited. In addition, as objectives were so varied, making comparisons between studies was difficult. The studies did however consider spinal stabilisation, at least in a broad sense, and the following insights were provided.

McGill and Kippers (1994) [15] suggested that an insight into interaction between sub-systems can be found by examining the transfer of moment from active to passive tissues at the limits of forward bending. Their investigation concluded that although electrically silent during full flexion, paraspinal muscles continue to provide elastic resistance via passive stretching. They suggest that this silence is an indication of the cessation of input from the central nervous system, likely as a result of some sort of active or passive tissue feedback. As the study was based on regional spinal measurements, nothing more than generalised theories could be extrapolated. In agreement with McGill and Kippers and again highlighting a requirement for inter-vertebral data, Arjmand et al. (2010) [41] showed that in both models, increased abdominal coactivity was predicted at the end of forward flexion. This mechanism is proposed by both studies to counterbalance moments in addition to the contributions of paraspinal muscles (i.e., passive resistance) and spinal ligaments.

In agreement with these studies, Paquet et al. (1994) [53] suggested that increased paraspinal activity permits the transmission of forces via these muscles, and is a mechanism to protect damaged passive structures. It was proposed that the alteration in hip-spine movement pattern in those with an absent FRP, may be a strategy to protect the lumbar spine near its maximum range (i.e., near its peak bending moment). This raises the importance of being able to measure kinematics in different regions of a chain (i.e., not just the lumbar region). Callaghan and Dunk (2002) [43] found that FRP was not present in the TES muscle during bending. As the study did not measure thoracic angular ROM however, and assuming cascading segmental flexion, some thoracic movement will have been expected to occur before the onset of movement in the lumbar region. It is therefore difficult to comment on deactivation mechanisms. However, the results do support the common conclusion in FRP studies that as passive tissues are stretched, they eventually reach a point at which they can counter the moment produced by bending the lower back. In this case, as flexion moment may be expected to be less during slumped sitting than standing flexion, the passive tissues are able to support the moment produced at a smaller lumbar angle. This is as much detail as the authors provided, and so it was not possible to relate their findings to interactions between systems or feedback mechanisms.

The study of Hashemirad et al. (2009) [45] was based on the idea that flexibility is linked to characteristics of the active and passive tissues. The authors suggested that in agreement with Panjabi’s hypotheses, when the CNS contends with increased flexibility in the passive tissues, it responds by increasing the contribution of the active system. This mechanism is represented in the study by the increased paraspinal activity associated with increased participant flexibility. The authors go on to suggest that such a mechanism is likely a spinal stabilisation strategy, however without inter-vertebral information this claim is difficult to support.

Generally speaking therefore, increased muscle activity is proposed as a mechanism that increases spinal stability, the review did however provide some contrasting findings. Peach et al. (1998) [35] investigating healthy controls, found a lack of co-contraction of abdominal and paraspinal muscles during flexion. This could suggest therefore that this may be an optimal stabilisation strategy employed by healthy spines, and that different activation strategies seen in LBP groups could represent adaptation mechanisms. In this case no speculation was provided regarding sub-system interactions. This is in contrast to the findings of Cholewicki et al. (1997) [44], who showed that trunk flexor and extensor co-activation was present during dynamic sagittal movement in participants with no history of low back pain. The study however only considered approximately 20° of flexion (i.e., around the neutral position) and cannot be compared directly with studies such as Peach et al. (1998) [35] where full flexion was performed. The authors again conclude that the co-activation is a neuromuscular activation strategy to increase stability of the lumbar spine. As a regional kinematic study, it was not possible to extrapolate insights into system interactions at the motion segment level, however the results do support the theory that any loss of spinal stiffness as a result of passive tissue damage, can be compensated by an overall increase in trunk muscle activation. As such, muscle activity may be useful as a clinical indicator. Further work was suggested which would benefit from investigations at the inter-vertebral level.

The findings of Sanchez-Zuriaga et al. (2015) [26] suggested that paraspinal activity was increased irrespective of the lumbar range of flexion achieved and may therefore indicate that deactivation mechanisms are not purely related to mechanisms such as the degree of ligament deformation as suggested elsewhere [21]. Burnett et al. (2004) [42] suggested that the LBP group in their study may have an underlying motor control dysfunction, either as a response to, or predisposing factor to a lumbar strain associated with the increased lower lumbar flexion and decreased local stabiliser activity. This is of course in direct contrast to the results of FRP studies considered in this review, which suggest that LBP is reflexively related to the increased activity of the paraspinals (i.e., the absence of the FRP). The authors also suggest that examining regions of the lumbar spine (e.g., upper and lower lumbar spine) is more revealing than measuring ranges of motion over the entire lumbar spine, given the contrast in kinematic behaviours found between groups in terms of lumbar regions. In agreement Dankaerts et al. (2009) [7] who also divided the lumbar spine into regions, concluded that increased muscle activity (examples found in both FP and AEP groups) likely represent maladaptive motor control strategies that potentially act as catalysts for ongoing strain and pain production, increase spinal load and result in impeded recovery. No detail about the proposed mechanisms were provided, however the value of further dividing kinematic regions (i.e., upper and lower lumbar spine) was demonstrated as the measurement of lumbar spine angles (i.e., T12-S2) and regional lumbar spine angles (e.g., lower lumbar spine L3-S2) produced distinctly different results.

The study by Kaigle et al. (1998) [20] was unique in that it was the only study reviewed with the capacity to comment on subsystem interactions at a motion segment level. In agreement with the theory that ligaments stretched in full flexion provide afferent impulses that then inhibit paraspinal muscles (Floyd and Silver 1955) [14], the authors conclude that as the patient group showed comparatively reduced inter-vertebral movement, the ligamentous mechanoreceptors were not sufficiently stimulated to provoke muscular inhibition. Unfortunately, due to a small sample size, even this study may not provide an inter-vertebral insight that can be generalizable to the wider population, which arguably is required to advance understanding in this area. Indeed, whilst the study of Arjmand was only small (n = 1), one of the key conclusions was that multi-joint kinematics combined with paraspinal EMG recordings would improve modelling accuracy.

Alternatively, O’Sullivan et al. (2006) [17] discussed their findings in relation to global and local paraspinal activity. The study showed that TES activity was extremely variable in participants during bending, a finding the authors suggested may be as a result of its role as a global muscle. As a globally acting muscle, it was argued to have more potential for variation in motor pattern, as it is was not directly responsible for local stabilisation as is the case for LMU. It may also be that the increase in TES activity is a strategy to maintain stability when LMU activity decreases, a mechanism perhaps employed to avoid excessive loading as a result of contraction Granata and Orishimo (2001) [9], or as additional resistance to the moment of flexion provided by the passive structures. In addition, Lariviere et al. (2000) [6] showed that TES muscles likely compensate for LES muscles when less active (such as during FRP). The authors suggest therefore it is likely that TES muscles have an important role to play in LBP patient motor control strategies, and so consideration of thoracic muscle activity should perhaps be given, even when investigations are focussed on dynamic movement within the lumbar spine.

### 4.3. Can the Information Aquired by Combining Lumbar Kinematic and Muscle Activity Measurements during Functional Movements Assist in Distinguishing between Groups of Healthy Controls and Those with Low Back Pain?

The review would suggest that there are many studies that have found distinguishing features in LBP populations (e.g., increased paraspinal muscle activity; decreased sagittal ROM), however, generally the study populations were small, and the large variations in methodology (particularly EMG placement and kinematic recordings) makes further analysis (including meta-analysis) difficult. There were also studies however that showed contrasting findings, or that were not able to distinguish between LBP and non-LBP groups. The wide range of methodological approaches makes it difficult to generalise such findings beyond the specific populations involved, which is a major limitation of research in this field. Whilst recommendations for EMG recordings and processing have been standardised [40,54], it would be of value if the muscles selected for these types of study were measured consistently from the same anatomical reference point. Likewise, in terms of the measurement of regional kinematics, it would be beneficial if such measurements were also standardised (i.e., between universally agreed landmarks such as L1-S1 to represent the lumbar region for example). Table 4, shows that in no two studies were the EMG electrode locations the same, and likewise all kinematic measurements differered in some way. The review does however highlight the potential of some variables for this purpose. As an example, Kienbacher et al. (2016) [47] using root mean square EMG amplitude, and regional measurements, showed that neuromuscular activation and kinematics can distinguish between CNSLBP patients with impaired or unimpaired muscle activation strategies. They suggest that the aging process is a stronger facilitator of this neuromuscular activity (i.e., increased paraspinal activity) than the pain associated with the condition. This the authors attribute to a likely increased excitability of the motor neurone pool associated with increased age [47].

The influence of pain on EMG and kinematic measurements is not well understood. It could be argued therefore that studies should either focus on healthy participants to gain a better understanding of what is normal [35], or use LBP groups that are pain free at the time of study, in order to remove for the influence of pain. In the O’Sullivan et al. (2006) [17] and Callaghan and Dunk (2002) [43] studies, both investigated low back pain free populations, and therefore the disagreement in their results (i.e., the existence or absence of the FRP in the TES) is most likely explained by methodological differences. The authors also suggest however that as TES activity is highly variable between individuals, this could possible represent inherently different motor control strategies. In addition to O’Sullivan’s findings (where no thoracic kinematic data was available), Nairn et al. (2013) [50] measured thoracic movement and showed that the deactivation of the TES during slumped sitting was related to increased angles of the thoracic segment movement. This supports the view that the decrease in activity is somehow related to stretch feedback of the ligaments, and the authors concluded that regional information was therefore important. In agreement, Luhring et al. (2015) [16] argued that the global approach (i.e., global trunk angle) was less preferable to the local approach (i.e., lumbar angle) as the mechanism of FRP is proposed to be dependent on local lumbar structures. This is a logical conclusion to make, and in continuation it is likely preferable still to obtain inter-vertebral information that relates directly to the lumbar structures involved.

### 4.4. Are There Opportunities to Improve Understanding of Sub-System Interactions and Low Back Pain Using Studies That Utilise Kinematic and EMG Measurements Concurrently?

The argument for the increased utilisation of inter-vertebral measurements when measuring spinal ROM and muscle activity concurrently, was alluded to frequently in this review. Inter-vertebral measurements would be important in this field, as this is the level at which spinal control feedback mechanisms are believed to be initiated [21]. Whilst regional kinematics are valuable, their measurements may best be related to globally acting musculature (e.g., muscles that span between the thoracic cage and the pelvis) [55], and so insights into stabilisation mechanisms resulting from ligamentous stress, or muscle spindle activation (i.e., related to segmentally acting tissues) would arguably be best provided by inter-vertebral data.

To collect kinematic information at this level however, presents some methodological problems. Whilst skin surface markers can be used to measure inter-vertebral motion, skin movement artifacts have been shown to result in poor reliability [56,57]. Reliable segmental data therefore typically requires more invasive techniques such as x-rays [58,59] or fluoroscopy [60,61,62,63,64], or as shown in this review the surgical insertion of intra-osseous pins [20]. Of these, fluoroscopy perhaps stands out, as it has been repeatedly demonstrated to be accurate and reliable [33,60,65,66,67], and as such may be the preferable option for future studies investigating interactions between sub-systems at the motion segment level.

The term nonspecific low back pain (NSLBP) by its definition, alludes to the fact that heterogeneity in LBP causes, can make it difficult to explain with any accuracy why kinematic or muscle activity parameters may differ between and within low back pain and non-low back pain groups. The key reason for increased muscle activity and decreased spinal ROM of motion provided by this review were likely adaptive mechanisms, related to spinal stabilisation. It is however difficult to demonstrate definite links between these parameters and LBP, due to the large number of possible pathoanotomical causes. A possible next step could be to investigate ROM and muscle activity in LBP populations that have been sub classified in some way. O’Sullivan et al. (2005) used a multidimensional classification system (MDCS) which included sub-groups of patients whose LBP was aggravated by flexion or extension [68]. An opportunity therefore, would be to investigate muscle activity and kinematic patterns at the motion segment level in LBP in patients who have been allocated to such groups, possibly providing new insight into the biomechanical origins of LBP at this level.

### 4.5. Key findings and recommendations

- Increased muscle activity and co-contraction are strategies adopted to stabilise the lumbar spine.
- Whilst generalised conclusions regarding spinal stabilisation were seen throughout the literature, insights into the understanding of spinal sub-system interactions at the motion segment level were limited.
- Parameters shown to distinguish between non-LBP and LBP populations include spinal ROM and trunk muscle activation, including the FRP. Typically, LBP groups demonstrated comparatively reduced ROM, increased muscle activity and an absent FRP.
- Future studies should consider more frequent use of sub-divided regional or inter-vertebral kinematic measurements, and would benefit from methodological standardisation.
- More extensive exploration of thoracic kinematic and muscle activity parameters may be beneficial in order to enhance understanding of lumbar spinal stabilisation mechanisms.

### 4.6. Limitations

Whilst the review focused on studies that investigated bending in the sagittal plane, it is acknowledged that other planes of movement (i.e., coronal and transverse) and different tasks may also provide important insights into spinal stabilisation mechanisms. In addition, as the quality assessment of each paper was performed by one individual, the repeatability between separate reviewers was not known. It is also acknowledged that the small number of data bases used to search for articles (i.e., PubMed and the Cochrane library) may be considered a limitation.

## 5. Conclusions

Many studies found differences in kinematic or EMG variables capable of distinguishing between LBP and healthy control groups, however the differences in methodology between studies mean that only broad generalisations can be made.

No one study set out with the explicit objective to explore sub-system interaction, however many did attempt to relate their findings to such mechanisms. A common weakness in study design was that studies used regional kinematic measurements, which can only ever at best provide a broad interpretation of sub-system interaction. It was therefore unsurprising that conclusions relating to sub-system interaction were limited. The studies that did were those that investigated sub-divided spinal regions or inter-vertebral kinematic measurements [7,20], and even these did not use truly inter-vertebral data, as the data was pooled from several inter-vertebral levels.

There is an apparent unmet need to better understand spinal stability and the assertion that the passive, active and motor control systems need to act in concert for function to be optimal [1]. An enhanced understanding could feasibly result in improved sub-grouping and diagnosis of LBP patients, and the development of more targeted therapeutic interventions, and therefore represents an important area of research. Whilst this review provides many examples of how changes in one sub-system may result in changes in another to compensate, the investigations have typically focused on regions of the spine and not at the motion segment level. In order to improve understanding of such interactions and the mechanisms behind them, it could be argued therefore that more emphasis could be placed on research focusing at the segmental level, the level at which communication between sub-systems is believed to be initiated. Improved understanding may also be hindered due to the fact that studies either focus on sub-systems individually or that it has not been possible to study their interactions during dynamic tasks.

It has been shown that although it is possible to measure numerous variables relating to spinal function, until one can measure in vivo inter-vertebral dynamic kinematics and relate it to one of the other sub-systems in detail, it will not be possible to make significant progress in this area. This lack of progression was reflected in this review and highlights the requirement for new approaches to research that incorporate these elements. Future studies should consider technologies that enable inter-vertebral measurements, not just in the lumbar spine but ideally throughout the thoracic, pelvic, hip and cervical regions too. It has been shown that stabilisation during sagittal bending can be influenced by the paraspinal muscle activity of both lumbar flexors and extensors, and abdominals, and that the TES may play an important role in lumbar stabilisation [5,25]. Measurement of these muscles, including activation timings and amplitudes, should therefore be included in studies whenever possible. Standardisation of investigation methodologies is also recommended, as the current heterogeneity in approaches makes any comparison between studies difficult.

## Figures and Tables

**Table 1:** Quality index assessment scores (* Studies that did not compare healthy controls to a low back pain group were rated using a 9 point scale instead of 10).

Quality Check	Category										Score	
Authors (year)	1	2	3	4	5	6	7	8	9	10	Score (/9 * or /10)	Score (%)
Arjmand et al. (2010) [41]	0	1	0	1	1	0	0	N/A	1	0	4 *	44
Burnett et al. (2004) [42]	1	1	0	1	1	1	1	1	1	1	9	90
Callaghan and Dunk 2002 [43]	1	0	0	1	1	0	1	N/A	1	1	6*	67
Cholewicki et al. (1997) [44]	1	1	0	1	1	1	0	N/A	1	1	7 *	78
Dankaerts et al. (2009) [7]	1	1	1	1	1	1	1	1	1	1	10	100
Hashemirad et al. (2009) [45]	1	1	0	1	1	1	0	N/A	1	1	7 *	78
Hay et al. (2016) [46]	0	1	0	1	1	0	0	1	1	1	6	60
Kaigle et al. (1998) [20]	1	1	1	1	1	1	0	0	1	1	8	80
Kienbacher et al. (2016) [47]	1	1	1	1	1	1	1	1	1	1	10	100
Lariviere et al. (2000) [6]	1	1	1	1	1	1	1	0	1	1	9	90
Liu et al. (2011) [48]	1	1	0	1	1	1	0	0	1	1	7	70
Luhring et al. (2015) [16]	1	1	1	1	1	1	0	N/A	1	1	8 *	89
Mayer et al. (2009) [49]	1	1	0	1	1	1	0	1	0	1	7	70
McGill and Kippers 1994 [15]	1	1	0	1	1	1	0	N/A	1	1	7 *	78
Nairn et al. (2013) [50]	1	1	0	1	1	0	1	N/A	1	1	7 *	78
Neblett et al. (2003) [51]	1	1	1	1	1	1	0	N/A	1	1	8 *	89
Ning et al. (2012) [52]	1	1	0	1	1	0	0	N/A	1	1	6 *	67
O’Sullivan et al. (2006) [17]	1	1	1	1	1	1	1	N/A	1	1	9 *	100
Paquet et al. (1994) [53]	1	1	1	1	1	1	0	1	1	1	9	90
Peach et al. (1998) [35]	1	1	0	1	1	1	0	N/A	1	1	7 *	78
Sanchez-Zuriaga et al. (2015) [26]	1	1	1	1	1	1	0	1	1	1	9	90

**Table 2:** Electromyography (EMG) quality assessment scores (* Studies that did not require normalisation were rated using a 3 point scale instead of 4) as per Abboud et al. (2017) [13].

EMG Quality Check	Category												Score	
Authors (year)	1.1	1.2	1.3	2.1	2.2	2.3	2.4	3.1	3.2	3.3	3.4	4	score (/3 * or /4)	score (%)
Arjmand et al. (2010) [41]	0	1	0	0	1	0	0	1	1	1	0	1	2	50
Burnett et al. (2004) [42]	1	1	0	1	1	0	0	1	1	1	0	1	4	100
Callaghan and Dunk 2002 [43]	0	1	0	0	1	0	0	1	1	1	0	1	2	50
Cholewicki et al. (1997) [44]	0	1	0	0	1	0	0	1	1	1	1	1	2	50
Dankaerts et al. (2009) [7]	1	1	1	0	1	1	1	1	1	1	1	1	4	100
Hashemerad et al. (2009) [45]	1	1	1	0	1	1	1	1	1	1	1	1	4	100
Hay et al. (2016) [46]	0	1	1	0	0	0	0	1	1	1	1	N/A	2 *	67
Kaigle et al. (1998) [20]	0	0	0	0	1	1	0	1	1	1	1	N/A	2 *	67
Kienbacher et al. (2016) [47]	1	1	1	0	1	1	1	1	1	1	1	1	4	100
Lariviere et al. (2000) [6]	0	1	0	1	0	1	0	1	1	1	0	N/A	2 *	67
Liu et al. (2011) [48]	0	0	N/A	1	0	1	0	1	1	1	1	1	3 *	100
Luhring et al. (2015) [16]	1	1	1	0	1	0	0	1	1	1	1	1	3	75
Mayer et al. (2009) [49]	0	0	0	0	0	0	0	1	1	1	0	0	1	25
McGill and Kippers 1994 [15]	1	1	1	0	1	0	0	1	1	1	1	1	3	75
Nairn et al. (2013) [50]	1	0	1	1	1	0	1	1	1	1	1	1	4	100
Neblett et al. (2003) [51]	1	1	0	1	1	0	0	0	0	0	1	N/A	2 *	67
Ning et al. (2012) [52]	0	0	1	0	1	0	0	1	1	1	1	1	2	50
O’Sullivan et al. (2006) [17]	1	1	1	1	1	1	1	1	1	1	1	1	4	100
Paquet et al. (1994) [53]	0	0	0	1	0	0	0	0	1	1	1	0	1	25
Peach et al. (1998) [35]	1	1	1	0	1	0	0	1	1	1	1	1	3	75
Sanchez-Zuriaga et al. (2015) [26]	1	1	1	0	1	1	1	1	1	1	1	1	4	100

**Table 3:** Combined quality index and EMG quality scores.

Authors (Year)	Quality Index Score (%)	EMG Quality Score (%)	Combined Score (%)
Arjmand et al. (2010) [41]	44	50	47
Burnett et al. (2004) [42]	90	100	95
Callaghan and Dunk 2002 [43]	67	50	58.5
Cholewicki et al. (1997) [44]	78	50	64
Dankaerts et al. (2009) [7]	100	100	100
Hashemerad et al. (2009) [45]	78	100	89
Hay et al. (2016) [46]	60	67	63.5
Kaigle et al. (1998) [20]	80	67	73.5
Kienbacher et al. (2016) [47]	100	100	100
Lariviere et al. (2000) [6]	90	67	78.5
Liu et al. (2011) [48]	70	100	85
Luhring et al. (2015) [16]	89	75	82
Mayer et al. (2009) [49]	70	25	47.5
McGill and Kippers 1994 [15]	78	75	76.5
Nairn et al. (2013) [50]	78	100	89
Neblett et al. (2003) [51]	89	67	78
Ning et al. (2012) [52]	67	50	58.5
O’Sullivan et al. (2006) [17]	100	100	100
Paquet et al. (1994) [53]	90	25	57.5
Peach et al. (1998) [35]	78	75	76.5
Sanchez-Zuriaga et al. (2015) [26]	90	100	95

**Table 4:** Study characteristics (*N* = 21).

Authors	Study Aim	EMG Variable and Lumbar Paraspinal Muscles Recorded (LMU = Lumbar Multifidus, LES = Lumbar Erector Spinae, TES = Thoracic Erector Spinae)	Lumbar Kinematic Measurements	Study Findings	Participants	Analysis
Arjmand et al. (2010) [41]	To compare a single joint model to kinematic driven model during trunk flexion.	Normalised EMG activity. Muscles Longissimus (3 cm lateral to L1) Iliocostalis (3 cm lateral to L3) Multifidus (2 cm lateral to L5).	Optotrak 4 camera system (regional) Lumbar region LED’s placed on pelvis and T12.	In both models, global extensor activity peaked around 30° of flexion, due to the increase in contribution of passive structures at this point. Extensors became silent between 50–70°.	*N* = 1 A male participant with no recent history of LBP.	Quantitative comparison was not performed.
Burnett et al. (2004) [42]	To determine whether differences exist in spinal kinematics and trunk muscle activity in cyclists with and without NSCLBP.	EMG activity was quantified by obtaining the mean activation, during a 5 crank revolution period. Muscles TES (5 cm lateral to T9) LMU (2–3 cm lateral to L4–L5).	3-Space Fastrak (regional) Lower lumbar L3 relative to S2 Upper lumbar T12 relative to L3.	The LBP group demonstrated greater lower lumbar flexion than controls associated with a loss of multifidus co-contraction.	*N* = 18 mean age 37.6 years 9 non low back pain 9 NSCLBP.	Independent sample *t*-tests.
Callaghan and Dunk 2002 [43]	To determine if FRP occurs in seated and slumped postures.	Ensemble average normalised EMG activity. Muscles TES (5 cm lateral to T9) LES (3 cm lateral to L3).	3-Space ISOTRAK (regional) Lumbar region Sacrum relative to L1.	FRP was shown in the TES, but not the LES during Slumped sitting. TES silence during sitting also happened at earlier angle of lumbar flexion than during standing.	*N* = 22 low back pain free participants 11 males mean age 21.3 years 11 females mean age 21.9 years.	Three way ANOVA, and Tukey’s post hoc multiple comparisons.
Cholewicki et al. (1997) [44]	To test the hypothesis that the flexors and extensors of the trunk are co-activated around a neutral spine posture.	Normalised EMG activity. Muscles TES (5 cm lateral to T9) LES (3 cm lateral to L3) LMU (2 cm lateral to L5–L5).	The use of 2 pieces of string attached to a chest harness and two potentiometers (regional).	Co-activation of trunk flexors and extensors was shown in healthy participants around a neutral posture.	*N* = 10 low back pain free participants 8 males and 2 females mean age 27 years.	A two factor repeated measures ANOVA.
Dankaerts et al. (2009) [7]	To test the ability of a model to distinguish between flexion pattern (FP) and active extension pattern (AEP) subgroups and healthy controls using lumbar kinematics and trunk muscle activity.	Normalised EMG activity. Superficial LMU (at the level of L5 orientated by a line between the PSIS and the L1–L2 interspace. Iliocostalis lumborum pars thoracis (lateral to L1).	3-Space Fastrak (regional) Upper lumbars T12 relative to L3 Lower lumbars L3 relative to S2.	Differences in muscle activity and spinal kinematics during flexion suggest that 2 distinct motor control patterns can exist in CNSLBP patients.	*N* = 67 participants 34 low back pain free controls, mean age 32 20 Flexion pattern NSLBP patients, mean age 36 13 Extension pattern NSLBP patients, mean age 40.	ANOVA and *post hoc* Bonferroni.
Hashemirad et al. (2009) [45]	To investigate the relationship between lumbar spine flexibility and LES activity during sagittal flexion and return.	Normalised EMG amplitude and signal onset/offset. Muscle LES (4 cm lateral to L3–L4).	Estimated using a camera and markers placed at the spinous processes of T12, L3 and S2 (regional).	During bending the ES of participants with high toe touch score deactivated at greater trunk and hip angles. Those with high modified Schober scores deactivated later and reactivated sooner in accordance with lumbar angle.	*N* = 30 low back pain free participants.	Pearson correlations and multiple linear regression analysis.
Hay et al. (2016) [46]	To show that wavelet coherence and phase plots can be used to provide insight into how muscle activation relates to kinematics.	EMG amplitude (linear envelope). Muscle Lumbar erector spinae (no details of positioning).	Oqus 400 motion capture system (regional) Reflective markers placed over T12 and S1.	The study showed good agreement between lumbar kinematics and linear enveloped sEMG. Validating the use of the wavelet coherence technique.	*N* = 14 low back pain free male participants.	The coefficient of determination (R²).
Kaigle et al. (1998) [20]	To concurrently quantify muscle activation of LES with the kinematics of lumbar motion segments, in low back patients and controls.	Root mean square (RMS) sEMG amplitude. Muscle LES (3 cm lateral to L3–L4).	A linkage transducer system secured by interosseous pins to L2-L3, L3-4 and L4-L5 motion segments (inter-vertebral).	ROM was less in low back pain patients and FRP occurred in participants when IV-ROM was complete before full trunk flexion	*N* = 13 6 low back pain free participants, mean age 40. 7 low back pain patients with suspected lumbar instability, mean age 51.	Wilcoxon rank-sum test and Wilcoxon matched-pairs signed rank test.
Kienbacher et al. (2016) [47]	To determine whether lumbar extensor activity and flexion relaxation ratios could differentiate low back pain patients (of various age groups) during flexion-extension task.	Normalised RMS sEMG amplitudes. Muscle LMU (lateral to L5) a line joining the iliac crests, and 2–3 cm bilateral and distal from their middle).	3-D accelerometers placed at the levels of T4 and L5. Used to calculate hip, lumbothoracic and gross trunk regions. (regional).	The sEMG activation was highest in over 60′s and female groups during standing. This possibly relates to why this group showed minimal changes during flexion. This group also demonstrated the highest hip, and lowest lumbothoracic angle changes.	*N* = 216 low back pain patients. 62 (60–90 year olds) 84 (40–59 year olds) 70 (18–39 year olds).	ANOVA and bootstrap confidence intervals.
Lariviere et al. (2000) [6]	To evaluate the sensitivity of trunk muscle EMG waveforms to trunk ROM and low back pain status during flexion-extension tasks.	Mean normalised EMG activity. Muscles LES and TES (exact locations not specified).	Video cameras and reflective markers. Trunk angles relative to the vertical plane were used to determine trunk flexion (A line between the hips and the centre of C7-T1) (regional).	Principal component analysis (PCA) distance measures were sensitive to trunk ROM but not low back status. The usefulness of PCA as an effective clinical tool was not established.	*N* = 33 15 low back pain patients, mean age 40 18 low back pain free participants, mean age 39.	ANOVA and ICC’s.
Liu et al. (2011) [48]	To develop a new test based on lumbar sEMG activity (the sEMG coordination network analysis approach) during flexion-extension, to distinguish between healthy control and low back pain groups.	Normalised RMS sEMG activity. Muscles An sEMG electrode array placed over the lumbar region (16 electrodes, target muscles not specified).	30° of trunk flexion, measured by a protractor (no further details) (regional).	Group network analysis shows a loss of global symmetric patterns in the low back pain group.	*N* = 21 11 low back pain patients, mean age 40. 10 low back pain free participants, mean age 28.	Did not specify. (However, groups comparison statistics and symmetry scores were used).
Luhring et al. (2015) [16]	To determine a kinematic measurement that best determines the onset and offset of the FRP.	Normalised sEMG onset and cessation. Muscle LES (4 cm lateral to L3).	Vicon MX motion capture camera system. Reflective markers placed at various locations throughout the spine including T12, L5 and pelvis (regional).	Lumbar kinematic measurements are preferential when the FRP is considered clinically.	*N* = 20 low back pain free participants, mean age 24.	Coefficients of Variation (CV) and ICC’s.
Mayer et al. (2009) [49]	To determine when FRP occurs in patients and to correlate the findings with lumbar ROM.	Mean RMS sEMG with pre-determined cut-off values. Muscles Not identified within paper.	Gross lumbar, hip/pelvic ROM using an inclinometer (no further details provided) (regional).	After a functional restoration program, both normal FRP and normal lumbar ROM were restored in the majority of patients.	*N* = 134 30 low back pain free participants, mean age 38. 104 low back pain patients (mean age not provided).	Descriptive statistics including mean and SD. Sensitivity and specificity. P-values and Odds ratios (not specified).
McGill and Kippers 1994 [15]	To examine the tissue loading during the period of transition between active and passive tissues during flexion.	Normalised sEMG activity. Muscles TES (5 cm lateral to T9) LES (3 cm lateral to L3).	3-Space Isotrak (regional) with sensors placed over the sacrum and T10.	The deactivation of lumbar extensor muscles during FRP occurs only in an electrical sense as they still provide force elastically.	*N* = 8 low back pain free participants, mean age 26.	Dynamic modelling.
Nairn et al. (2013) [50]	To quantify slumped sitting both in terms of spinal kinematics and sEMG.	Mean normalised sEMG activity. Muscles Lower TES (5 cm lateral to T9) LES (4 cm lateral to L3) LMU (Adjacent to L5 orientated along a line between the PSIS and the L1–L2 interspinous space.	Vicon motion capture camera system. Reflective markers placed at various locations throughout the spine including T12, L1 and bilateral PSIS’s (regional).	During slumped sitting lower sEMG activity was found in the thoracic and lumbar erector spinae compared to upright sitting. Patterns varied depending on the degree of bending at each area of the spine. Thoracic kinematic and EMG information is therefore useful in these type of studies	*N* = 12 low back pain free participants, mean age 23.	ANOVA and Bonferroni correction.
Neblett et al. (2003) [51]	To assess EMG activity in terms of the FRP during dynamic flexion and to determine whether abnormal FRP patterns in NSLBP patients can be normalised.	RMS sEMG cut-off values. Muscles LES (2 cm lateral to L3).	Inclinometers at T12 and the sacrum (regional).	In asymptomatic participants, the flexion relaxation (FR) angle was always less than the maximal voluntary flexion (MVF) angle. Of the patients that completed a functional restoration program, 94% achieved FR compared to 30% pre-treatment.	*N* = 66 12 low back pain free participants, mean age 34. 54 chronically disabled work-related spinal disorder (CDWRSD) patients	Descriptive statistics for ROM and FRP
Ning et al. (2012) [52]	To determine a boundary at which the passive tissues begin to take a significant role in trunk extensor moment (and therefore at what point EMG assisted modelling is no longer valid).	Normalised EMG activity. Muscles LES at two levels (3 cm lateral to L3 and 4 cm lateral to L4).	A magnetic-field based motion tracking system with sensors placed at T12 and S1. Lumbar flexion calculated as the pitch of T12 relative to S1 (regional).	EMG-assisted models should consider the action of the passive tissues at lower flexion angles than previously thought.	*N* = 11 low back pain free participants, mean age 26.	ANOVA and Tukey–Kramer post-hoc testing
O’Sullivan et al. (2006) [17]	To investigate the FRP of spinal muscles in healthy participants during slumped sitting from an upright position.	Normalised EMG activity offset. Muscles TES (5 cm lateral to T9) LMU (Adjacent to L5 orientated along a line between the PSIS and the L1–L2 interspinous space.	3- Space Fastrak with sensors placed over T6, T12 and S2. (regional).	LMU is active during neutral sitting and demonstrates FRP when moving from upright to slumped sitting. FRP of these muscles is also different to when standing. More variation was found in EMG patterns of the TES.	*N* = 24 low back pain free participants, mean age 32.	ANOVA and ICC’s
Paquet et al. (1994) [53]	To compare healthy controls and low back pain patients in terms of hip-spine movement interaction and EMG, and to verify the relationships between kinematics and EMG in these groups.	Raw EMG envelope. Area under the curve and ratio of activity at different parts of the flexion-extension cycle (not-specified). Muscles LES (at the level of L3, distance not-specified).	Electro goniometers measured angular displacements at the hip and lumbar spine using landmarks of T8 and S1 (regional).	LES activation patterns were found to be significantly different between groups when flexion was performed at the same rate and range. Abnormal hip-spine movement related to an absence of the FRP at full flexion.	*N* = 20 10 low back pain free participants, mean age 34. 10 low back pain patients, mean age 38.	Mann-Whitney U test and Kruskal-Wallis test
Peach et al. (1998) [35]	To document the lumbar kinematics and trunk EMG activation patterns of healthy controls during tasks including sagittal flexion	Mean normalised EMG. Muscles TES (5 cm lateral to T9) LES (3 cm lateral to L3) LMU (1–2 cm lateral to L5).	3-Space Isotrak with sensors placed over T12 and Sacrum. (regional).	A database of normal lumbar spinal kinematics and EMG patterns was created for future reference against LBP groups.	*N* = 24 low back pain free participants, mean age 22.	Descriptive statistics, ANOVA and Tukey’s honestly significant difference (HSD) post-hoc testing
Sanchez-Zuriaga et al. (2015) [26]	To compare healthy controls and LBP patients in terms of lumbopelvic kinematics and erector spinae activity	Mean normalised EMG activity, and start and end of FRP. Muscle LES (3 cm lateral to L3).	A 3-dimensional videophotogrammetric system, with markers placed at T12, L3, L5 and the sacrum (regional).	During pain free periods, recurrent LBP patients showed significantly greater LES activity during flexion and extension. Lumbar ROM and FRP were not found to be useful to distinguish between groups.	*N* = 30 15 low back pain free participants, mean age 41. 15 patients with recurring low back pain (currently in a pain free stage), mean age 45.	Mann-Whitney U test
